# Platelet-Derived Drug Targets and Biomarkers of Ischemic Stroke—The First Dynamic Human LC-MS Proteomic Study

**DOI:** 10.3390/jcm11051198

**Published:** 2022-02-23

**Authors:** Karolina Gawryś, Aleksandra Turek-Jakubowska, Jakub Gawryś, Maciej Jakubowski, Janusz Dębski, Ewa Szahidewicz-Krupska, Małgorzata Trocha, Arkadiusz Derkacz, Adrian Doroszko

**Affiliations:** 1 Department of Neurology, 4th Military Hospital, Weigla 5, 50-556 Wroclaw, Poland; karolina.maria.gawrys@gmail.com (K.G.); lek.aturek@gmail.com (A.T.-J.); 2 Department of Internal Medicine, Hypertension and Clinical Oncology, Faculty of Medicine, Wroclaw Medical University, Borowska 213 Str., 50-556 Wroclaw, Poland; jakub.gawrys@umw.edu.pl (J.G.); ewa.szahidewicz-krupska@umw.edu.pl (E.S.-K.); arkadiusz.derkacz@umw.edu.pl (A.D.); 3 Lower Silesian Centre for Lung Diseases, Grabiszyńska 1 Str., 53-439 Wroclaw, Poland; maciekjak@gmail.com; 4 Institute of Biochemistry and Biophysics, Polish Academy of Sciences, Pawińskiego Str., 02-106 Warsaw, Poland; jasio.ibb@gmail.com; 5 Department of Pharmacology, Faculty of Medicine, Wroclaw Medical University, Chałubińskiego Str., 50-345 Wroclaw, Poland; malgorzata.trocha@umw.edu.pl

**Keywords:** stroke, platelet proteome, biomarkers, platelets, thymidine phosphorylase 4, proteomics, drug target, human

## Abstract

(1) Objective: The aim of this dynamic LC-MS (liquid chromatography and mass spectrometry) human platelet proteomic study was to identify the potential proteins candidates for biomarkers of acute ischemic stroke (AIS), their changes during the acute phase of stroke and to define potential novel drug targets. (2) Methods: A total of 32 patients (18–80 years old) were investigated that presented symptoms of AIS lasting less than 24 h from the onset, confirmed by neurological examination and/or new cerebral ischemia visualized in the CT (computed-tomography) scans. The analysis of platelet proteome was performed using LC-MS at baseline, and then on the third and seventh day from the onset of symptoms. The control group was demographically matched without any clinical signs of acute brain injury. (3) Results: The differences between platelets, at 24 h after first symptoms of stroke subjects and the control group included: β-amyloid A4 and amyloid-like protein 2, coactosin-like protein, thymidine phosphorylase 4 (TYMP-4), interferon regulatory factor 7 (IRF7), vitamin K-dependent protein S, histone proteins (H2A type 1 and 1-A, H2A types 2B and J, H2Av, -z, and -x), and platelet basic protein. The dynamic changes in the platelet protein concentration involved thrombospondin-1, thrombospondin-2, filamin A, B, and C. (4) Conclusions: This is the first human dynamic LC-MS proteomic study that differentiates platelet proteome in the acute phase of ischemic stroke in time series and compares the results with healthy controls. Identified proteins may be considered as future markers of ischemic stroke or therapeutic drug targets. Thymidine phosphorylase 4 (TYMP-4) holds promise as an interesting drug target in the management or prevention of ischemic stroke.

## 1. Introduction

Cardiovascular disease (CVD) remains among the most common causes of morbidity and mortality, as reported worldwide annually. In Europe, almost half of fatal cases have been directly connected with CVD [1]. Stroke is among the most common causes of death and persistent disability in adults [2]. In 70–80% of cases, it is caused by cerebral ischemia [3] and is increasingly referred to as “acute cerebral syndrome”, indicating its similarity to acute coronary syndromes. Therefore, the detection of biochemical markers of vascular-derived brain damage should have a diagnostic and prognostic meaning, similar to that of myocardial infarction and heart failure (e.g., troponin (Tn) and N-terminal natriuretic peptide type B (NT-proBNP)). Since biomarkers must be characterized by certain features [4,5], there is still a lack of substances that could be used as reliable indicators of acute central nervous system (CNS) ischemia, which could allow quick performance of diagnostics and appropriate treatment [6].

Platelets play a pivotal role in the pathogenesis of cardiovascular disease, including ischemic stroke [7], and are an important therapeutic target in the treatment and secondary prevention of cardiovascular events. Thrombocytes can be affected by numerous stress factors present in the blood, which lead to their activation and aggregation that trigger a prothrombotic cascade. During activation, they secrete numerous compounds that support initiation or exacerbation of ischemia [8]. Proteomics enable the search for differences between protein content and could provide information about their changes during development of a disease and its treatment [9]. Since a stroke is associated with thrombus formation, investigating changes in platelet protein expression from subjects in the acute phase of cerebral ischemia should provide important information regarding its pathophysiology as well as provide some useful biomarkers and drug targets.

Hence, in this study, we assessed the dynamic variability of platelet proteome and peptidome in the acute phase of ischemic stroke in order to define its new platelet-derived biomarkers or drug targets with neuroprotective potential. The aim of the study was to falsify the hypothesis regarding the absence of significant differences in the platelet proteome between patients with ischemic stroke as compared with a control, using a liquid chromatography and mass spectrometry (LC-MS) technique. The dynamic time-dependent relationships between the course of the disease and the level of identified potential candidate proteins were also analyzed.

## 2. Materials and Methods

### 2.1. Recruitment

**Figure 1:**
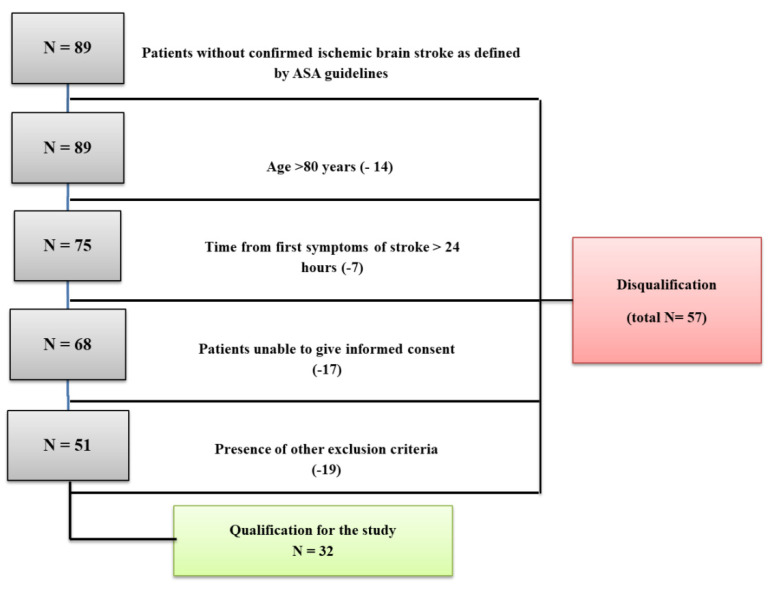
A flow chart presenting the recruitment of the subjects.

**Figure 2:**
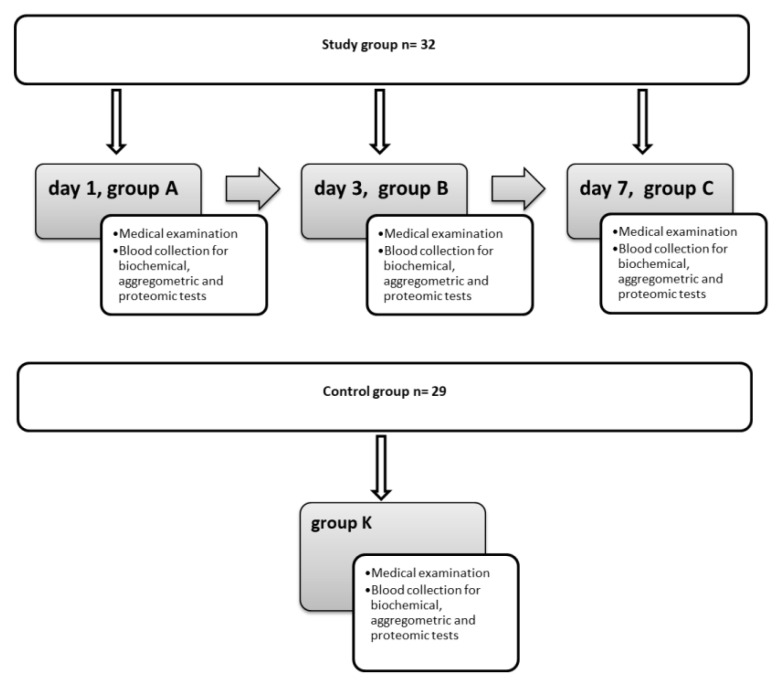
Study protocol.

A total of 61 subjects with newly diagnosed acute ischemic stroke (AIS) qualified for the study: 32 patients, aged 18–80 years, hospitalized for this reason at the Clinical Department of Neurology, and 29 volunteers, demographically matched to the study group, hospitalized at the Clinical Department of Internal Medicine of the same hospital. Participants of the study group met all the inclusion criteria defined by the study protocol (Figure 1 shows a flow chart of the study and Figure 2 presents the experimental protocol).

The exclusion criteria included: inability to obtain fully informed consent, no accurate history of past or coexisting diseases, lack of precise information on the used pharmacotherapy and the duration of CNS ischemia symptoms, qualification for thrombolytic treatment or thrombectomy, anemia or thrombocytopenia. Additionally, subjects were excluded with a history of vascular diseases of the nervous system, extensive head injuries, atrial fibrillation, cancer, chronic inflammatory diseases, active infections, chronic kidney disease (eGFR < 45 mL/min/1.73 m^2^), and the use of drugs potentially affecting the results of study (antithrombotic, anticonvulsants, type 5 phosphodiesterase inhibitors, contraceptives, and hormone replacement therapy).

### 2.2. Study Design

Within 24 h after the onset of the first stroke symptoms, the patients underwent a detailed anamnesis, physical examination, and full evaluation by a neurologist under the current guidelines [10]. The diagnosis was supported by an imaging of the central nervous system using a Siemens^®^ SOMATOM Definition 64-row dual-source CT scanner. Biological material (40 mL of venous blood) was non-traumatically collected (using a Sarstedt^®^ S-Monovette aspiration and vacuum kit) three times from subjects qualified for the study group: on the first (group A), third (group B), and seventh (group C) day of hospitalization, and from the control group once on the day of obtaining consent to participate in the project (group K) (Figure 2). From the collected material, the aggregation tests and evaluation of platelet proteome using liquid chromatography with mass spectrometry (LC-MS) were performed. Moreover, blood serum was used to conduct biochemical tests (creatinine, eGFR, sodium, potassium, hsCRP, glucose, thyroid-stimulating hormone, bilirubin, hepatic enzymes, and lipid profile) in order to evaluate the cardiovascular risk, following the 2016 European Society of Cardiology (ESC) guidelines [11]. Whole blood was obtained to determine the complete blood count (CBC) and plasma was used to determine coagulation parameters. The biochemical tests, coagulation, and CBC were performed in a certified hospital laboratory using the following analyzers: Sysmex^®^ (XT-4000i), Siemens^®^ (The Dimension^®^ EXLTM), and Thermo Fisher Scientific^®^ (Konelab 20 Clinical Chemistry Analyzer).

### 2.3. Platelet Preparation

Whole blood with sodium citrate was supplemented with a solution of PGI2 prostacyclin (PGI2 in Tris-Cl buffer with pH 9.0 at a concentration of 1 mg/1 mL) at a concentration of 0.06 μg/mL of whole blood, which prevented spontaneous platelet aggregation. To obtain platelet-rich plasma (PRP), the solution was centrifuged for 20 min at 230× *g*. PGI2 at 0.3 µg/mL concentration was added to PRP and centrifuged at 1000× *g* for 10 min at 21 °C to obtain low platelet plasma and platelet sediment. Then, plasma was removed, and the remaining platelets were washed three times with 1 mL of Tyrodes HEPES buffer (H-T buffer), Ca^2+^ free, with pH 7.4. After the last rinsing, the obtained precipitate was supplemented with H-T buffer pH 7.4 to the volume of 3 mL. The obtained suspension was analyzed for platelet count and contamination with white blood cells (WBCs) and red blood cells (RBCs) in a Sysmex^®^ device (Clinical Laboratory Department, University Hospital, Wroclaw, Poland). The pure platelet (PLT) suspension was brought with Tyrodes HEPES pH 7.4 buffer to a final concentration of 2.5 × 10^8^/mL and centrifuged at 16,000× *g* for 10 min at 4 °C. Then, the material was stored at −80 °C in Eppendorf^®^ tubes until proteomic determinations were performed in the Environmental Mass Spectrometry Laboratory operating at the Institute of Biochemistry and Biophysics of the Polish Academy of Sciences (IBB PAS).

Within 24 h after the onset of the first stroke symptoms, the patients underwent a detailed anamnesis.

### 2.4. LC-MS Proteomic Analysis

The LC-MS analysis was performed at the Environmental Mass Spectrometry Laboratory of IBB PAS. Platelet proteins were extracted by incubation of platelet deposit in 1% sodium deoxycholate buffer solution in 25 mM ammonium bicarbonate, and then sonification in a water bath (10 cycles of 30 s) was performed to dissolve the proteins. Then, the suspension was centrifuged at 14,000× *g* for 15 min (Eppendorf Minispin). The obtained protein solution was reduced with 50 mM phosphine (TCEP) (30 min, 60 °C), alkylated with 200 mM thiosulfonate (MMTS) (15 min, at room temperature), and digested with trypsin overnight (modified trypsin sequencing, ega V5111). In the morning, the digestion was stopped by acidifying the samples with 2 μL 10% trifluoroacetic acid (TFA) and the precipitated sodium deoxycholate was removed by centrifugation. Then, the concentration of the obtained peptides was estimated using direct detection (Millipore^®^).

The resulting peptide mixtures were applied to an RP-18 pre-column (nano-ACQUITY Symmetry C18, Waters 186003514), as a mobile phase 0.1% TFA was applied in water. Then, the solution was applied to an RP-18 HPLC nano-column (nanoACQUITY BEH C18, Waters 186003545), using acetonitrile (5–35% AcN in 180 min) in the presence of 0.1% formic acid, with a flow rate of 250 nL/min. The column outlet was directly connected to the Velos Orbitrap ion source (Thermo Electron Corp., San Jose, CA, USA) with a change from MS (peptide mass measurement) to MS/MS (peptide fragmentation). To ensure the absence of cross-contamination by the previous samples, each time, empty tests were analyzed first. Subsequently, the data were processed by Mascot Distiller to verify hits in the Swiss-Prot database (29119124) limited to the Homo sapiens sequence [12]. Peptides with a Mascot Score above a threshold value corresponding to <1% FDR were identified as positive. The determined proteins were analyzed using the Diffprot^®^ software [13].

### 2.5. Statistical Analysis

The statistical analysis was performed using the Statistica 13.3 StatSoft^®^. The presented data are expressed as an arithmetic mean with standard deviation (SD). The Mann–Whitney U-test or the Student’s *t*-test, following the Shapiro–Wilk test and Levene’s test as appropriate, were used to assess the significance of differences between the mean values and ANOVA followed by Tukey’s test, or a Friedman test was used when more than two groups were investigated. The analysis of proteomic/peptidomic data were performed using the Diffprot^®^ software (by the laboratory performing the determination), as described in the previous section.

### 2.6. Bioethics Statement

All procedures in the study protocol were investigated and approved by the Local Bioethical Committee (Approval number: KB—371/2018). Participants, after learning about study procedures, signed a written informed consent which was previously verified by the bioethical committee. The project is consistent with the principles of the Declaration of Helsinki (Seventh Revision, 64th World Medical Association meeting, Fortaleza, 2013).

## 3. Results

### 3.1. Demographic, Clinical, and Biochemical Characteristics of the Investigated Population

There were no differences in the basic demographic characteristics nor co-morbidities between groups, including hypertension, coronary artery disease, diabetes mellitus (Table 1). None of the subjects had chronic kidney disease with egfR < 45 mL/min, as the CKD was an exclusion criterion in this study. In the CBC, significantly lower mean platelet volume (MPV) and significantly higher mean corpuscular hemoglobin mass index (MCH), white blood cells (WBCs), and neutrophil concentration were observed in subjects in the acute phase of ischemic stroke. The biochemical analysis in the acute stroke group (A) showed significantly higher values of TSH and glycemia followed by significantly lower serum potassium concentration as compared with the control group, however, all of these values were within the normal range.

### 3.2. Proteomic Analysis

During the examination of platelet proteomes, we identified several proteins with concentrations that differed significantly among groups (Table 2). The differences between platelets 24 h after first symptoms of stroke and the control group included:

1. Beta-amyloid A4 protein and amyloid-like protein 2;
2. Coactosin-like protein;
3. Thymidine phosphorylase TYMP-4;
4. Interferon regulatory factor 7;
5. Vitamin K-dependent protein S;
6. Histone proteins (H2A type 1, H2A type 1-A, H2A type 2B, H2A J, and H2Av, -z, -x);
7. Platelet basic protein.

The differences in the concentrations of interferon regulatory factor 7 (IRF7) and thymidine phosphorylase TYMP-4 persisted for the next three days from the onset of stroke (a significant difference between group B and the control).

The dynamic changes in platelet protein concentration in the course of stroke involved:

1. Filamin A, filamin B, filamin C (group A vs. group B, following the first two days from the onset of the stroke)
2. Thrombospondin-1, thrombospondin-2 (group A vs. group C, following one week from the onset of symptoms).

It is noteworthy for both thrombospondin types that significant changes in their concentrations were also observed between control and the stoke subjects at one week from the onset of symptoms.

## 4. Discussion

This is the first human dynamic LC-MS proteomic study to evaluate the changes in human platelet proteome and peptidome in the acute phase of ischemic stroke using a highly reproducible LC-MS technique.

### 4.1. Demographic and Clinical Characteristics

The study groups were similar regarding age, sex, as well as occurrence of co-morbidities including hypertension, coronary artery disease, and diabetes mellitus. The higher WBC and neutrophiles in the complete blood count could be attributed to the acute phase reaction in the first 24 h following the onset of stroke.

The higher plasma glucose levels on admission in the stroke group could reflect a non-fasting condition or sympathetic activation on admission, as the prevalence of diabetes was similar in both groups. Nevertheless, assessment of the HbA1c in both groups, which would clarify this issue, was missing in some subjects, therefore, it was not considered in this study.

Some studies have indicated that the level of thyroid stimulating hormone may be associated with the occurrence and prognosis of an acute stroke. Moreover, elevated TSH levels in subclinical hypothyroidism did not increase the risk of stroke and were likely to be associated with a better prognosis after an ischemic event, especially if the hypothyroidism appeared before the onset of the disease. Nevertheless, the serum TSH concentration in the acute ischemic stroke as a single parameter is difficult to interpret, as it is strongly dependent on a patient’s age [14,15,16,17].

### 4.2. Proteomic Analysis

Platelet secretion of thromboxane A2, adenosine diphosphate (ADP), matrix metalloproteinase-9 (MMP-9)—following their activation—promotes thrombus formation in a positive feedback loop, resulting in uncontrolled secretion of preformed platelet proteins and peptides from platelet cytosol and granules. The platelet releasate contains the secreted inflammatory and vasoactive molecules, including granules or micro-vesicles; α-granules are the most abundant and contain both the membrane-bound proteins as well as soluble proteins. Thrombosis and mechanisms of platelet-mediated inflammation require close interaction of platelets with endothelial and immune cells, as well as with the extracellular matrix. Activation of the endothelial cells, local hypoxia, and subsequent lactic acidosis promote thromboinflammation. Membrane proteins are expressed and comprise integrins, adhesive glycoproteins, and other granule membrane-specific receptors. The content of α-granules secreted through special surface-connected channels, open canalicular system (OCS) and SNARE is the core of the fusion machinery. Proteomic studies have suggested that hundreds of soluble proteins are released by α–granules from activated platelets and many of them are present in plasma with differences in structure or function [8,18,19]. Moreover, antiplatelet drugs have been demonstrated to modify the content of the platelet releasate, which could determine their modulative role on the platelet paracrine function, in addition to their anti-aggregatory function [20,21]. Hence, we believe that some of the identified proteins could be released from platelets in the acute phase of stroke in response to their activation and developing neuro-thromboinflammation.

As far as the literature is concerned, there are few reports describing platelet proteins in the acute phase of ischemic stroke (AIS). One of them, performed by Cevik et al., analyzed the difference in PLT proteome in blood collected within 24 h after occurrence of stroke symptoms as compared with a control group of the same size, and demonstrated the presence of 83 proteins that differed significantly between the groups [22]. In our study, the material obtained from 32 patients after the ischemic incident and 29 volunteers from the control group were analyzed. Additionally, the blood was collected not only at the first day of hospitalization, but also after three and seven days from the ischemic incident. Then, the results were compared in time intervals (first, third, and seventh days of hospitalization) within the study group. The identified proteins with concentrations that varied significantly are potential candidates for biomarkers of an acute ischemic stroke and may reflect the cascade of events related to its evolution over time. With this approach, the study provides a more accurate description of the cascade of changes in the platelet proteome as compared with those of previous studies.

In the current study, we observed an elevated concentration of intraplatelet β-amyloid protein (APP) in the AIS as compared with the control group. This is in line with some previous reports that showed the presence of β-amyloid protein in platelet α-granules, which could be a source of up to 90% of plasma circulating APP. Moreover, platelet activation has been shown to result in a three-fold increase in APP expression on their surface [23]. It has been shown that APP, via positive feedback, can induce platelet aggregation, thus, initiating a cycle based on continuous activation and its release from the platelets [24]. This theory was supported in a study by Ming Y. Shen et al., where β-amyloid stimulated the platelet signaling pathway leading ultimately to their aggregation [25]. The tests performed on gerbils showed the connection between increased accumulation of APP in neurons and their exposure to ischemia [26]. Given the above, an increase in β-amyloid protein plasma concentration could potentially be a useful indicator of the acute phase of ischemic stroke, but its broad role in the development of CNS degenerative diseases may significantly reduce the specificity of its determination [27]. However, as in the case of troponin determination in the acute phase of myocardial infarction, it may be clinically relevant to prove an increase or decrease in APP concentration as an indicator of CNS ischemia.

Analogous observations to those of APP in our study concerned the APP-like protein 2 (APLP-2), i.e., its intraplatelet concentration was elevated in the AIS as compared with the control group. Due to their common origin, they have been assigned similar functions: transport, intracellular signaling, and apoptosis [28]. In pathophysiology, they have been associated with the induction of trophic changes in neurons and synapses [29]. Because of the small amount of information on the family of amyloid-like proteins and their role in stroke, similarly as in the case of APP, further research is necessary to establish their exact role in this group of patients.

The proteomic analysis showed that the concentration of coactosin-like protein (COTL) was lower in the control group as compared with acute CNS ischemia. Its function is currently the subject of numerous studies. A correlation between decreased coactosin-like protein concentration or its absence in platelets and impaired glycoprotein GPIb function was shown, which could be directly translated into prolonged bleeding time and corresponding protection against blood clot formation. In addition, a reduction in coactosin-like protein levels resulted in a reduction in leukotriene formation, and consequent reduction in proinflammatory activity. Supporting this hypothesis, Inge Scheller et al. showed that the lack of this protein did not affect the number and activation, but it reduced the formation of platelet aggregates on collagen and their adhesion to vWF in in vitro tests [30]. It is possible that lowering platelet COTL concentration in patients with stroke could have a protective effect against further clot formation and aggravation of the ischemic changes. Considering the above characteristics, the possibility of using COTL as a stroke biomarker remains questionable, however, its function makes it a potential new drug target in primary and secondary AIS prevention.

Interestingly, we have shown dynamic changes in the expression of the thymidine phosphorylase TYMP-4 during ischemic stroke, which was significantly higher as compared with the controls. We postulate that TYMP-4 might become a platelet therapeutic target [31] in humans, reducing the extent of ischemic penumbra in the acute phase of ischemic stroke. Thymidine phosphorylase is also known as a platelet-derived endothelial growth factor, the presence of which has been described in human thrombocytes, monocytes, and macrophages (including astrocytes). This protein promotes angiogenesis in vivo, stimulates the growth and chemotaxis of endothelial cells in vitro, regulates platelet hemostasis [32], which in turn leads to the development of cardiovascular diseases, including ischemic stroke [33]. Under physiological conditions, TYMP-4, together with VEGF, are responsible for the continuity of the blood-brain barrier. Noteworthy, there are several reports that TYMP expression is increased in post-ischemic neurons and may protect them from ischemia-reperfusion injury. Recently, a growing body of evidence points at TYMP-4 as a potential drug target, and some animal studies have shown that its inhibition may provide a novel effective and safe therapeutic strategy. Since the TYMP-4 inhibitor molecule tipiracil is already being used in clinical practice (in the chemotherapy of colorectal cancer as an adjuvant that inhibits the disintegration and increases the concentration of the chemotherapeutic nucleoside analogue, trifluridine), we started the future direction of our study on the usefulness of TYMP-4 by tipiracil in reducing the extent of brain ischemia reperfusion injury in an animal model. Nevertheless, to the best of our knowledge, our study is the first to demonstrate the dynamic and time-dependent upregulation of platelet TYMP-4 in the acute phase of human stroke as a “real-life” clinical scenario, which translates the experimental results from bench to the bedside.

In the current study, a higher concentration of interferon regulatory factor 7, the key IFN type I transcription regulator, was observed in the platelet proteome of subjects with AIS as compared with the control. Statistically significant results were present when the control group was compared with the study group on the first and third day of hospitalization. There is only limited information available in the literature on the role of IRF-7 in ischemic stroke. Scientific reports have connected the occurrence of these incidents with the development of a proinflammatory response involving interferon regulatory factors. This response initiates locally at the site of ischemia, and then the inflammatory mediators spread throughout the entire body [34]. Moreover, in a study conducted on mice, Stevens et al. demonstrated that the presence of IRF-3 and IRF-7 was necessary to induce TLR-dependent (Toll-like receptors) neuroprotection. On this basis, these two factors were identified as key mediators in the neuroprotective genomic program, which ultimately led to reduced damage during ischemic stroke [35]. The reason for the higher concentration of IRF-7 in platelets in our study group still needs further explanation. This may be related to excessive use and requirement for interferon regulatory factor 7 in the acute phase of ischemia, which would be associated with its potential neuroprotective and proinflammatory function. Therefore, the elevated concentration of platelet IRF-7 may be be a biomarker that could be potentially useful for establishing a diagnosis of AIS, but also as a factor indicating better prognosis.

Some data have confirmed the relationship between reduced plasma concentration of protein S, a non-enzymatic element of hemostasis that regulates the activity of protein C, and the occurrence of ischemic stroke [36,37]. In studies conducted on mice, it was found that the injection of protein S had a neuroprotective function, reducing the size and extent of ischemic lesions [38]. There is a single report that has proved that platelet α-granules contained protein S, the release of which was triggered by the presence of thrombin. In addition, protein S could bind to the stimulated platelets, which consequently activated the anticoagulant activity of activated protein C on their surface [39]. In this study, we observed an increased concentration of protein S in platelet proteome in the acute phase of ischemic stroke as compared with the control. Based on previous reports, it can be considered that the expression of protein S increases in platelets during cerebral ischemia to activate its anticoagulative and neuroprotective properties. Therefore, it can be concluded that an increase in protein S concentration in platelets may indicate the presence of ischemic changes, as well as the occurrence of actions aimed at reducing the neurological deficit after a stroke. Determination of platelet protein S in AIS may not be useful to confirm the diagnosis, but the finding of its low concentration may be an indication to administer exogenous protein S to reduce ischemic penumbra.

We observed a decrease in the platelet concentration of filamins between the first and third day after an acute ischemic stroke. Filamins belong to the family of actin-binding proteins that serve as a scaffold for signaling proteins and connect to the cytoskeleton [40]. Among these peptides, there are three FLN isoforms, i.e., A, B, C, which come from three different homologous genes. It has been shown that FLN mutations in platelets can lead to impaired activation of integrin IIb3 or, on the contrary, by increasing its activity, can lead to an increased risk of thrombosis. It has also been found that they are involved in both the production and activation of platelets [41]. Due to the limited information available so far, it can only be speculated that changes in the concentration of filamins may be a potentially useful indicator of the acute phase of stroke, or that they are a consequence of the introduction of antiplatelet therapy. This issue requires further validation and clinical trials on a larger group of patients.

In this study, significant increases in TSP-1 and TSP-2 concentrations were observed on the seventh day of hospitalization. Thrombospondins play a variety of functions including participating in the aggregation of platelets, affecting endothelial cells and smooth muscles, and playing a role in regulating angiogenesis [42,43]; the earliest discovered is thrombospondin-1 (TSP-1), which is mostly released from the platelet α-granules following their activation [44]. The thrombospondin group has also been assigned an important role in the development and regeneration of nervous system cells [45]. In rat studies, the TSP was isolated from embryonic tissue. Moreover, an increase in its concentration was found after damage to the nervous tissue of the brain of these animals with cainic acid [46]. Based on the cited study, it can be concluded that this protein contributes both to the formation of nerve cells during embryonic development and their regeneration after pathological incidents. A study by Möller et al. showed an increase in thrombospondin concentration from four to seven days after a facial nerve injury, which overlapped the peak of the maximum post-traumatic proliferation of the microglia [47]. During an AIS, increased expressions of TSP-1 and TSP-2 at the level of mRNA and protein, which exhibited different profiles of temporal expression, were confirmed in a study by Navarro-Sobrino et al. [48], where increased thrombospondin-2 levels on admission in patients with ischemic stroke and increased thrombospondin-1 levels two hours after treatment with tissue plasminogen were identified. In addition, an ischemic stroke is followed by intensified angiogenesis, especially in the penumbra region, which also involves proteins from the thrombospondin group [49]. Based on the cited and our results, it can be postulated that TSPs have a protective function after the occurrence of a stroke and determination of their intraplatelet concentration may indicate the beginning of nervous tissue regeneration processes or the effectiveness of reperfusion treatment. In addition, the available literature also showed that these proteins can act as biomarkers for predicting a long-term prognosis. In a study by Gao et al., higher TSP-1 concentrations in stroke subjects were associated with a shorter six-month survival [50]. Nevertheless, there are reports that thrombospondins may exacerbate the negative effects by reducing tissue reperfusion. Isenberg et al. demonstrated that endogenous TSP-1 limited the recovery of tissue perfusion after ischemic damage in the skin lobes of mice [51]. In order to more precisely assess the roles of thrombospondin-1 and thrombospondin-2 and their effects on the prognoses in stroke patients, longer prospective observation following the onset of AIS is needed.

In the present study, increased concentrations of peptides belonging to the family of histone proteins were observed in platelets: H2A type 1 and H2A types 1-A, 2-B, J, and -v, -z, -x were observed in subjects with stroke. The basic role of histone proteins is the regulation of gene transcription. They connect with cellular DNA in nucleosomes, which are physiologically located in cellular nuclei [52]. Extracellular histone proteins, isolated from plasma, may originate from dying cells or be actively secreted during the inflammation. Their elevated concentration has been detected in inflammatory (including autoimmune), ischemic, and neoplastic diseases [53,54]. They can play a proinflammatory and toxic role, which was proven by Xu et al. [55]. Moreover, it has been shown that human histone proteins cause fibrinogen binding, the release of von Willebrand factor, increased P-selectin exposure, and exert a pro-aggregation effect [56]; among the proteins of this group the strongest prothrombotic functions are performed by H4 histone [57]. There are no other reports on the dynamics of changes in the concentration of histone proteins in platelets during an acute ischemic incident. Based on our data, it can be postulated that the concentrations of intraplatelet histones might be used as biomarkers of AIS, facilitating diagnosis and predicting disease processes associated with the activation of the immune system.

In our study, significantly higher concentrations of platelet basic protein (PBP) were observed in subjects with stroke, which was consistent with the results of studies by Rex et al. who proved an increased release of PBP during thrombin activation of platelets [58]. A few reports on platelet basic protein have indicated its multidirectional effects, i.e., it participates in the formation of inflammation; acts as a bactericide; and participates in angiogenesis, hemostasis, and clot formation [59,60]. PBP, after posttranslational modification, forms, among others, connective tissue activating peptide III (CTAP-III), neutrophil-1 activating peptide (NAP-2), β-thrombomodulin, and thrombocydine [61]. The available literature indicated that PBP had proinflammatory properties, while the closely related platelet factor 4 (PF4) played a role in hemostasis and thrombosis [62,63]. Based on the studies, it can be assumed that the demonstrated increase in the PBP concentration in platelets may be a consequence of ongoing pathological processes including ischemia and secondary inflammation. As this effect only slightly decreases over the first seven days after an AIS, changes in platelet PBP concentration may be a potentially useful indicator not only of the acute phase of ischemic stroke, but also may confirm a history of CNS ischemic incident in the more distant past. Further studies on a larger group of patients are needed to understand the exact role of PBP usefulness as an indicator of previous CNS ischemia.

## 5. Conclusions

In this first human dynamic LC-MS based study on ischemic stroke, several qualitative and quantitative differences in platelet proteomes were found as compared with the control group, as well as within the group of stroke subjects in the course of the acute phase of stroke. The identified proteins may act as potential biomarkers or indicators of a high risk of an ischemic incident. The changes in the platelet proteome observed in patients with ischemic stroke during hospitalization, point, in turn, to their relationship with the disease dynamics. These proteins may serve as the prognostic factors, indicating the timing of the ischemic episode and the patient’s prognosis, but may also help to determine the risk of stroke delivery. In addition, some of the proteins might be considered to be potential targets for future drugs with potential neuroprotective effects. Thymidine phosphorylase 4 (TYMP-4) holds promise to be an interesting drug target in the management or prevention of ischemic stroke, however, future studies are needed to demonstrate its exact role in the pathophysiology and pharmacological management.

## Figures and Tables

**Table 1:** Demographic, biochemical, and clinical characteristics of the stroke and control group.

	Stroke Group 1 Day of Hospitalization (A) (Mean ± SD)	Control Group (K) (Mean ± SD)	*p*
***n***	31	29	
**Age** (years)	62.7 ± 9.35	62 ± 11.4	>0.10
**F/M**	13/18	14/15	>0.10
**Hypertension**	21/10	17/12	>0.10
**Previous MI or PCI/CABG**	6/25	3/26	>0.05
**CKD**	0/31	0/29	>0.10
**DM**	8/23	6/23	>0.10
**Hb** (g/dL)	14.39 ± 1.63	13.78 ± 1.66	>0.10
**Ht** (%)	42.65 ± 4.45	40.87 ± 4.90	>0.10
**RBC** (10^6^/µL)	4.79 ± 0.59	4.68 ± 0.60	>0.10
**MCH** (pg)	30.44 ± 1.62	29.48 ± 1.95	<0.05 *
**MCV** (fL)	90.19 ± 4.69	87.38 ± 4.86	>0.10
**MCHC** (g/dL)	33.71 ± 0.83	33.73 ± 1.34	>0.10
**WBC** (10^3^/µL)	9.16 ± 3.48	7.03 ± 2.60	<0.001 *
**Lymphocytes** (10^3^/µL)	1.91 ± 0.68	2.01 ± 0.78	>0.10
**Neutrophils** (10^3^/µL)	6.49 ± 3.28	4.50 ± 2.46	<0.025 *
**PLT** (10^3^/µL)	220.57 ± 59.87	244.58 ± 46.99	>0.10
**MPV** (fL)	9.38 ± 1.26	10.87 ± 0.83	<0.005 *
**hsCRP** (mg/L)	4.98 ± 5.39	5.51 ± 6.00	>0.10
**Potassium** (mmol/L)	3.85 ± 0.34	4.14 ± 0.43	<0.001 *
**Sodium** (mmol/L)	138.77 ± 2.50	140.19 ± 2.43	>0.10
**Glucose** (mg/dL)	126.90 ± 48.35	109.87 ± 55.32	<0.005 *
**Creatinine** (mg/dL)	0.92 ± 0.26	0.89 ± 0.28	>0.10
**AST** (AU)	19.00 ± 7.06	23.35 ± 12.71	>0.10
**ALT** (AU)	21.63 ± 9.79	25.73 ± 14.82	>0.10
**Bilirubin** (mg/dL)	0.54 ± 0.07	0.73 ± 0.29	>0.10
**TCh** (mg/dL)	184.27 ± 48.20	203.38 ± 53.80	>0.10
**HDL** (mg/dL)	49.20 ± 13.6	56.57 ± 17.08	>0.10
**LDL** (mg/dL)	108.83 ± 41.5	122.81 ± 47.45	>0.10
**TG** (mg/dL)	124.28 ± 60.28	128.23 ± 61.20	>0.10
**TSH** (µIU/mL)	3.58 ± 2.68	1.53 ± 0.81	<0.025 *
**APTT** (s)	27.66 ± 3.59	28.73 ± 5.35	>0.10
**INR**	0.96 ± 0.07	0.99 ± 0.05	>0.10

SD—standard deviation; *—statistically significant *p*-value (*p* < 0.05); *p*—test probability; Hb—hemoglobin; Ht—hematocrit; F/M—female/male; MI—myocardial infarction, PCI—percutaneous coronary intervention; CABG—coronary artery y-pass graft CKD—chronic kidney disease with eGFR < 45 mL/min.; DM—diabetes mellitus; RBC—red blood cells; MCH—mean hemoglobin mass index in erythrocytes; MCV—mean red cell volume; MCHC—mean hemoglobin concentration in a unit volume of red cells; WBC—leukocytes; PLT—platelet count indicator; MPV—mean platelet volume; hs-CRP—high sensitivity C-reactive protein; AST—aspartate aminotransferase; ALT—alanine aminotransferase; TCh—total cholesterol; HDL—high density lipoprotein fraction; LDL—low-density lipoprotein fraction; TG—triglyceride concentration; TSH—thyrotropic hormone; APTTT—partial thromboplastin time after activation; INR—normalized prothrombin time.

**Table 2:** Comparison of the platelet proteins differing between the stroke (A, B, and C) vs. control group (K) as well as within the stroke subjects in the defined time intervals (A vs. B and A vs. C).

Protein	Compared Group	q Value	Ratio	Change Multiplier	Number of Peptides
**Beta-amyloid protein A4**	**K vs. A**	0.00836 *	**0.76**	**1.31**	**12**
	K vs. B	1	0.53	1.89	14
	K vs. C	1	0.48	2.07	14
	A vs. B	1	0.8	1.24	14
	A vs. C	1	0.87	1.15	14
**Amyloid-like protein 2**	**K vs. A**	0.00836 *	**0.76**	**1.31**	**12**
	K vs. B	1	0.53	1.89	14
	K vs. C	1	0.48	2.07	14
	A vs. B	1	0.8	1.24	14
	A vs. C	1	0.87	1.15	14
**Coactosin-like protein**	**K vs. A**	0.02739 *	**1.48**	**1.48**	**7**
	K vs. B	0.92572	1.55	1.55	7
	K vs. C	1	1.32	1.32	7
	A vs. B	1	1.11	1.11	7
	A vs. C	1	0.93	1.08	7
**Thymidine phosphorylase-4**	**K vs. A**	0.00041 *	**0.74**	**1.36**	**41**
	**K vs. B**	0.01351 *	**0.66**	**1.57**	**47**
	K vs. C	1	0.71	0.41	48
	A vs. B	1	0.99	1.01	48
	A vs. C	1	0.99	1.01	48
**Interferon regulatory factor 7**	**K vs. A**	0.00041 *	**0.74**	**1.36**	**41**
	**K vs. B**	0.01351 *	**0.66**	**1.57**	**47**
	K vs. C	1	0.71	0.41	48
	A vs. B	1	0.99	1.01	48
	A vs. C	1	0.99	1.01	48
**S protein dependent on vitamin K**	**K vs. A**	0.00836 *	**0.55**	**1.82**	**7**
	K vs. B	1	0.92	1.09	9
	K vs. C	1	0.92	1.09	9
	A vs. B	1	1.37	1.37	9
	A vs. C	1	1.12	1.12	9
**Filamine A** **Filamine B** **Filamine C**	K vs. A	1	0.96	1.04	211
	K vs. B	0.21428	1.13	1.13	238
	K vs. C	1	1.11	1.11	243
	**A vs. B**	0.04415 *	**1.15**	**1.15**	**239**
	A vs. C	0.98621	1.16	1.16	244
**Thrombospondin-1** **Thrombospondin-2**	K vs. A	0.94972	0.9	1.12	86
	K vs. B	1	0.8	1.24	98
	**K vs. C**	0.00061 *	**0.7**	**1.44**	**100**
	A vs. B	1	0.98	1.02	99
	**A vs. C**	0.02598 *	**0.86**	**1.16**	**100**
**Histone proteins**	**K vs. A**	0.04951 *	**0.16**	**6.12**	**2**
	K vs. B	1	0.68	1.47	2
	K vs. C	1	0.83	1.21	2
	A vs. B	1	4.19	4.19	2
	A vs. C	1	4.63	4.63	2
**Platelet basic protein**	**K vs. A**	0.00041 *	**0.62**	**1.62**	**12**
	K vs. B	0.08849	0.76	1.31	14
	K vs. C	0.12338	0.71	1.4	14
	A vs. B	1	0.93	1.08	14
	A vs. C	1	0.93	1.08	14

*—coefficient q statistically significant (q < 0.05); q—test probability; A—blood collected on day 1 of hospitalization; B—blood collected on day 3 of hospitalization C—blood collected on day 7 of hospitalization; K—blood collected from the control group; K vs. A—difference at the beginning of observation; K vs. B—difference after a three-day observation; K vs. C—difference after a seven-day observation; A vs. B—the difference between the first and third day of hospitalization; A vs. C—difference between the first and seventh day of hospitalization. The bold text highlighet in red presents the significant differences.

## Data Availability

The original data used to support the findings of this study are available from the corresponding author upon request.
